# Triple retinal arterial macroaneurysms in a hypertensive patient with hypothyroidism

**DOI:** 10.1186/s12886-023-02953-x

**Published:** 2023-05-10

**Authors:** Asmaa Mohammed Gamal El-Deen, Alzahraa Mohammed Gamal-edeen

**Affiliations:** 1 Department of Ophthalmology, Faculty of Medicine (for Girls), Al-Azhar University, Cairo, Egypt; 2 Department of Dermatology, Faculty of Medicine (for Girls), Al-Azhar University, Cairo, Egypt

**Keywords:** Multiple retinal arterial macroaneurysms, Hypertension, Optical coherence tomography angiography, Hypothyroidism, Acquired ichthyosis

## Abstract

**Purpose:**

To present the unique case of numerous, recurring retinal arterial macroaneurysms (RAMs) in a hypothyroid patient with hypertension.

**Methods:**

67-year-old woman’s clinical findings, laboratory results, fundus fluorescein angiography, optical coherence tomography (OCT), and optical coherence tomography angiography (OCTA) are given. Over a two-year period, the patient was monitored.

**Results:**

A 67-year-old woman presented to the outpatient clinic with vitreous and dense subretinal hemorrhages in her right eye. RAM rupture was discovered along the suprotemporal retinal arteriole. A diagnosis of systemic arterial hypertension was made. Two months later, the vitreous hemorrhage spontaneously resolved and the patient’s vision improved. After nine months of initial presentation, the patient developed another RAM. Meanwhile the patient developed ichthyosis caused by hypothyroidism. Because fundus fluorescein angiography revealed that the first RAM was still active, an intravitreal injection of anti-VEGF was administered six months afterwards. More proximal RAM with macular edema developed after another six months necessitating laser photocoagulation. However macular edema didn’t resolve and a second injection of intravitreal anti-VEGF was given.

**Conclusions:**

Patients with RAMs, particularly if multiple or recurring, should be thoroughly investigated and assessed, particularly for secondary causes of hypertension. OCT and OCTA are useful tools for RAM confirmation and follow-up. It is important to look into how RAM behavior interacts with coexisting macular edema, and other variables affecting hemodynamic status.

**Supplementary Information:**

The online version contains supplementary material available at 10.1186/s12886-023-02953-x.

## Introduction

RAMs are rare, acquired dilatations of the retinal arterial vasculature that usually occur within the first three branches of the arteriolar tree[1].

Hypertension and ageing cause vascular wall hyaline degeneration, loss of autoregulatory tone and elastic recoil, and arterial dilatation[2].

RAMs are typically discovered as unilateral, solitary lesions[3].They can occur along the same or different arteries of the same eye in up to 20% of cases[4]. A rare case of three RAMs along the superotemporal arteriole in a hypertensive patient due to hypothyroidism is presented.

## Case study

**Fig. 1:**
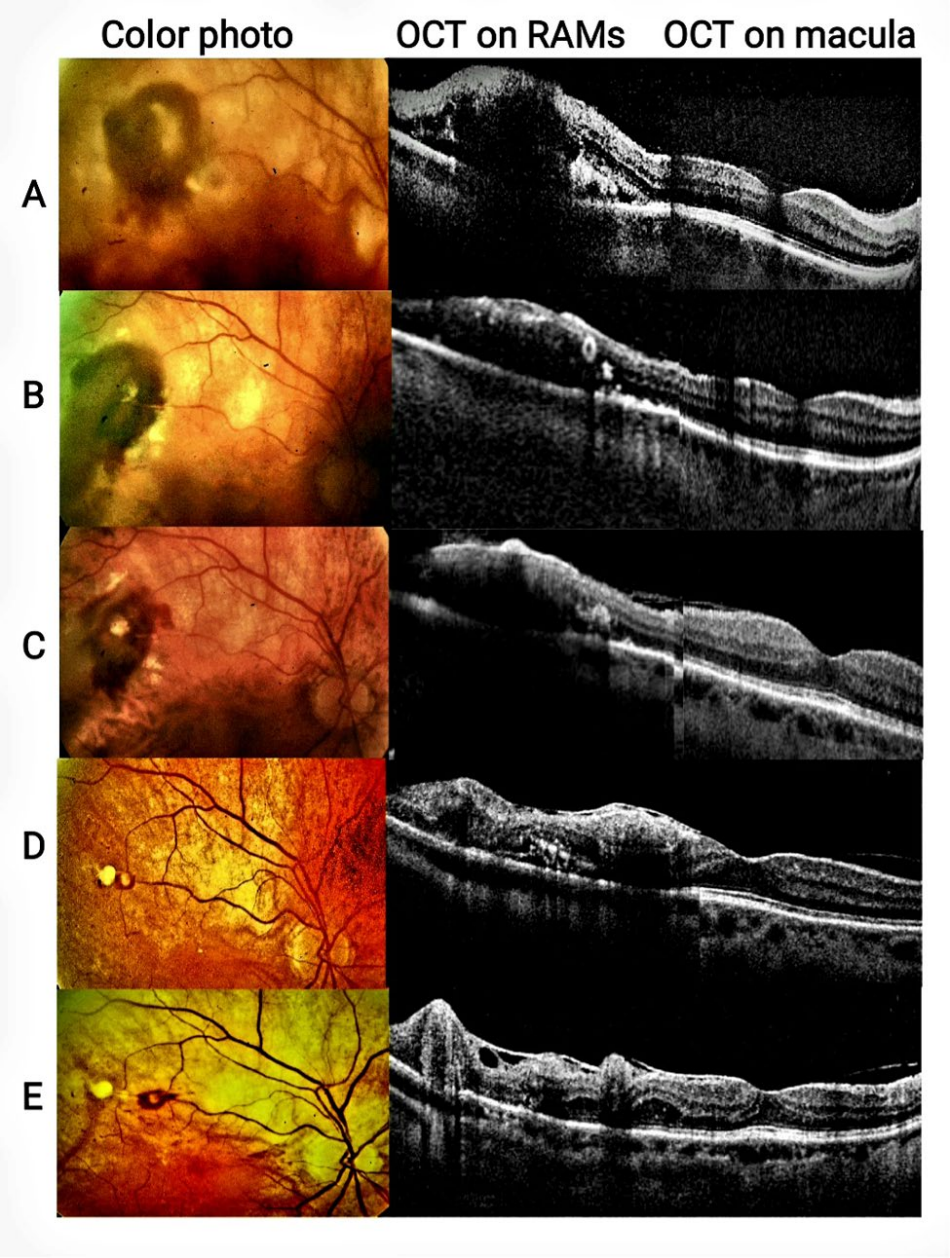
Composite images representing the chronological progress of RAMs lesions from left to right; Fundus photographs, OCT on RAMs lesions, and OCT on macula. (**A**) At presentation, Fundus photograph of the superotemporal RAM with surrounding exudation and dense subretinal hemorrhage and confirmed by OCT. BCVA was 20/125. (**B**) One week later (after oral Alphintern): pouring of blood out of subretinal hemorrhage. RAM lesion could be visualized patent on OCT. (**C**) Two weeks later; vitreous hemorrhage started to resolve. The patient was observed for a period of 2 months. The hemorrhage and edema resolved, and the visual acuity improved to 20/50. The patient remained stable for another 4 months and follow up was set after 3 months. (**D**) Second RAM lesion documented (9 months from initial presentation) and was exudative and observed for another 6 months. Six months later, BCVA was 20/40 and OCT confirmed edema thus Anti-VEGF was given. (**E**) After 6 months of anti-VEGF therapy, third lesion developed that was exudative with edema involving the macula, and the vision again decreased to 20/50. The decision was made to undergo laser photocoagulation around the RAM. Abbreviations: RAM, retinal artery macroaneurysm; BCVA: best corrected visual acuity; VEGF, vascular endothelial growth factor

A 67-year-old woman presented to our hospital’s outpatient clinic in January 2021 with newly onset of floaters and decreased vision in her right eye. Her best-corrected visual acuity (BCVA) at presentation was 20/125 in the right eye and 20/50 in the left. Slit-lamp examination revealed early cataracts and intraocular pressures within the normal range in both eyes. A vitreous hemorrhage, a dense subretinal hemorrhage, and surrounding exudates in the superior arcade were discovered during a fundus examination of the right eye **(**Fig. 1A**).** The left eye’s fundus examination was unremarkable. Optical coherence tomography (OCT) and optical coherence tomography angiography (OCTA ),using RTVue XR Avanti; AngioVue; optoVue, Inc, Fremont, California, USA, as well as fundus photography (by Topcon TRC-50EX fundus camera), were performed and confirmed the diagnosis of rupture RAM. Ultrasound imaging revealed vitreous and subretinal hemorrhages **(Supplementary Fig. 1**).

Fundus Fluorescein Angiography (FFA) could not be performed due to a lack of supplies during the pandemic. A systemic review revealed no associated systemic disease, and a cardiologist consultation revealed mild elevation of arterial blood pressure (BP = 150/90 and HR = 65 beats per minute). Before beginning antihypertensive therapy, a blood pressure chart was recommended. The patient’s arterial blood pressure was intermittently elevated (with the 24-hour BP average was 140/90), and antihypertensive medication was prescribed based on this chart. As a result of the RAM being away from the macula and the primary cause of the drop in vision being vitreous hemorrhage, the decision was made to observe for spontaneous involution. For vitreous hemorrhage, an anti-edematous enzyme (Alphintern®) was administered orally.

One week later, fundus photography revealed blood pouring from the subretinal hemorrhage into the vitreous cavity, prompting the patient to discontinue Alphintern®. The patient was kept on hypertension medication and was closely monitored **(**Fig. 1B**).**

Two months later, the vitreous hemorrhage had resolved, the RAM had spontaneously regressed with a BCVA of 20/25 **(**Fig. 1C**).** The patient was kept under observation and had follow-up exams every other month.

Nine months after her initial presentation, her BCVA had dropped to 20/80, and OCT and OCTA revealed the presence of a second RAM lesion along the same vessel that was more proximal than the first (Fig. 1D). Meanwhile, the patient developed multiple skin lesions on her forehead, forearm, and trunk, which were diagnosed as acquired ichthyosis by a dermatologist **(Supplementary Fig. 2**). Laboratory tests revealed severe hypothyroidism, with TSH > 100 uIU/ml and free T4 = 0.01 poml/l. The patient was referred to an endocrinologist, who prescribed Eltroxin Table 150 mcg and adjusted the dose based on her signs and lab results.

**Fig. 2:**
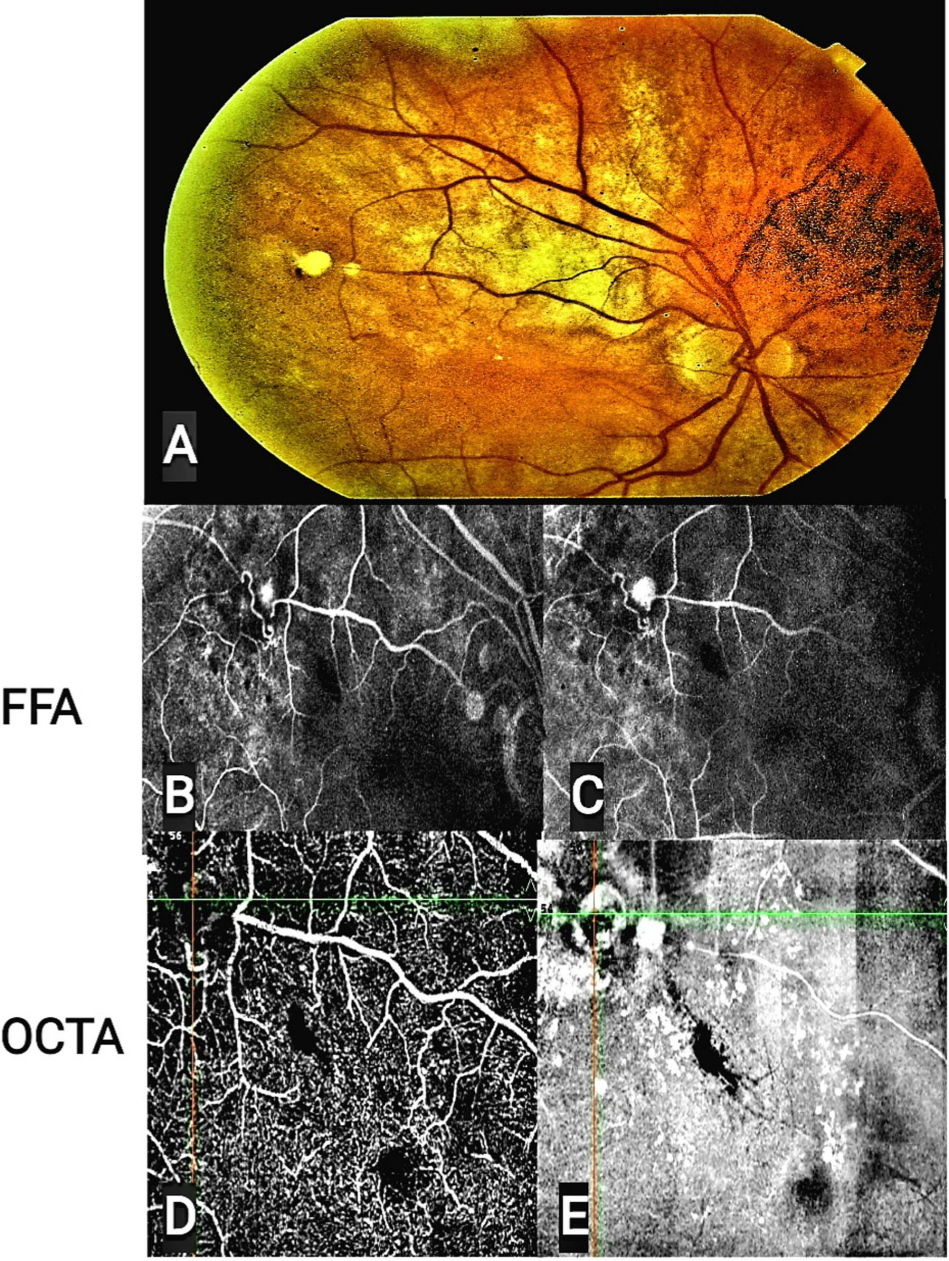
Composite images of fundus photography (**A**), FFA (**B**, **C**) and OCTA (**D**, **E**) of the patient fifteen months after initial presentation demonstrating two RAMs lesions. FFA showed hyperfluorescent lesion at superotemporal arteriole , corresponding to first RAMs, during arteriovenous filling (**B**) and increased fluorescence in late phase (**C**).OCTA flow (**D**)and enface (**E**)demonstrating patent first lesion and sclerosed second lesion with surrounding exudation. Abbreviations: RAM, retinal artery macroaneurysm, FFA: Fundus fluorescein angiography. OCTA: optical coherence tomography angiography

FFA revealed a regressed second lesion six months later, but the first lesion remained active with minimal leakage **(**Fig. 2**).** As a result, the patient received one intravitreal anti-VEGF injection (ranibizumab; LUCENTIS®).

**Fig. 3:**
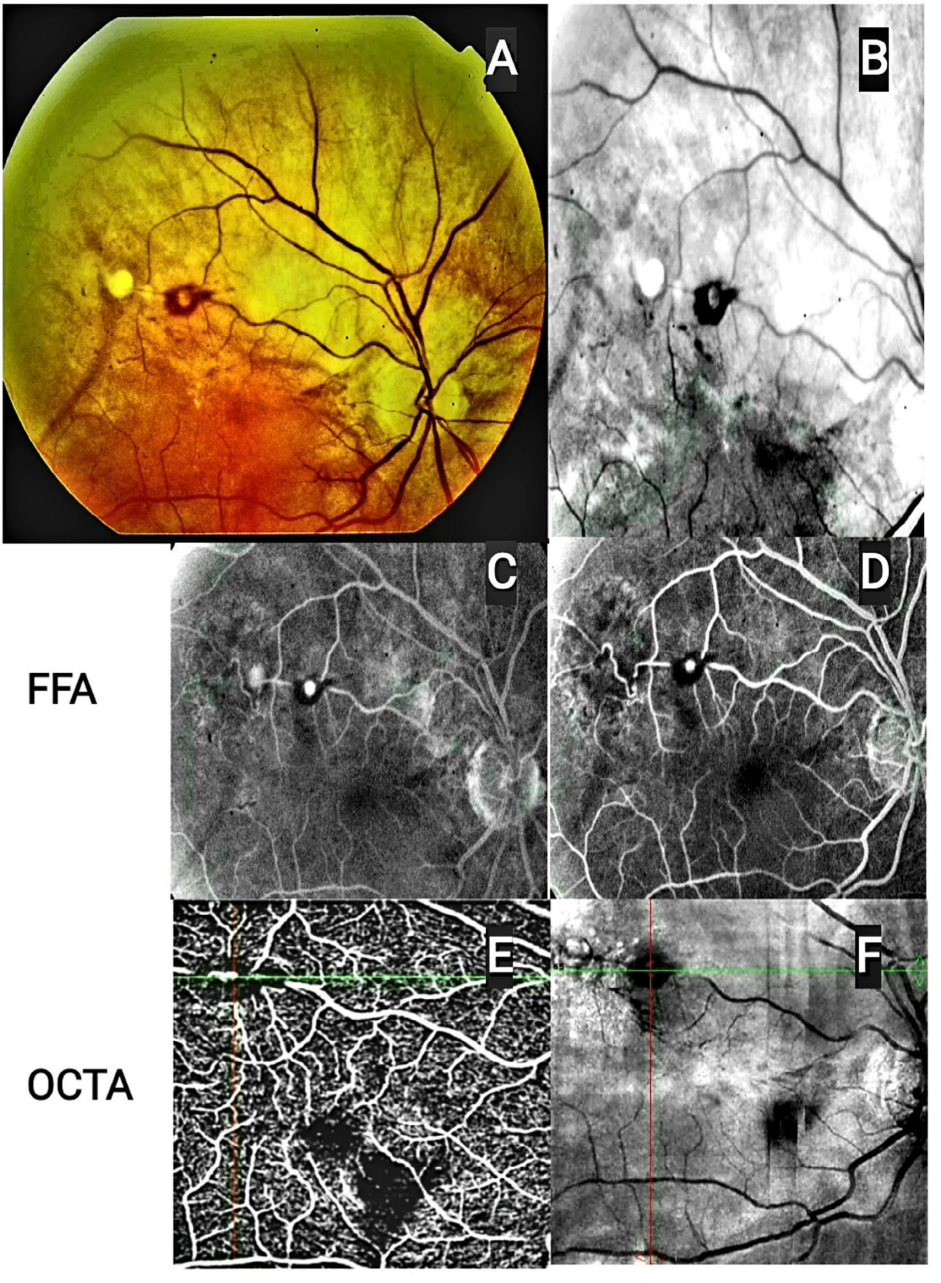
Composite images of fundus photography (**A**), red free (**B**), FFA (**C**, **D**) and OCTA (**E**, **F**) of the patient 21 months after initial presentation demonstrating the three RAMs lesions. FFA showed hyperfluorescent lesion at superotemporal arteriole ,corresponding to third RAM, during arteriovenous filling (**C**) and increased fluorescence in late phase with surrounding leakage and blockage by blood, a ring of alternating fluorescence could be detected at site of previous dense subretinal hemorrhage and late staining of sclerosed former RAM lesion (**D**). OCTA flow (**D**) and enface (**E**) demonstrating patent third lesion and sclerosed pervious two lesions with surrounding exudation. Abbreviations: RAM, retinal artery macroaneurysm, FFA: Fundus fluorescein angiography. OCTA: optical coherence tomography angiography

After another six months, a new third RAM lesion with surrounding hemorrhage and exudates appeared in the same artery proximal to the scars of the previous two lesions **(**Fig. 1E **and** Fig. 3**).** As the lesion and its exudates were closed to the macula, the patient was given a laser photocoagulation session and was scheduled for follow-up **(Supplementary Fig. 3).**

**Fig. 4:**
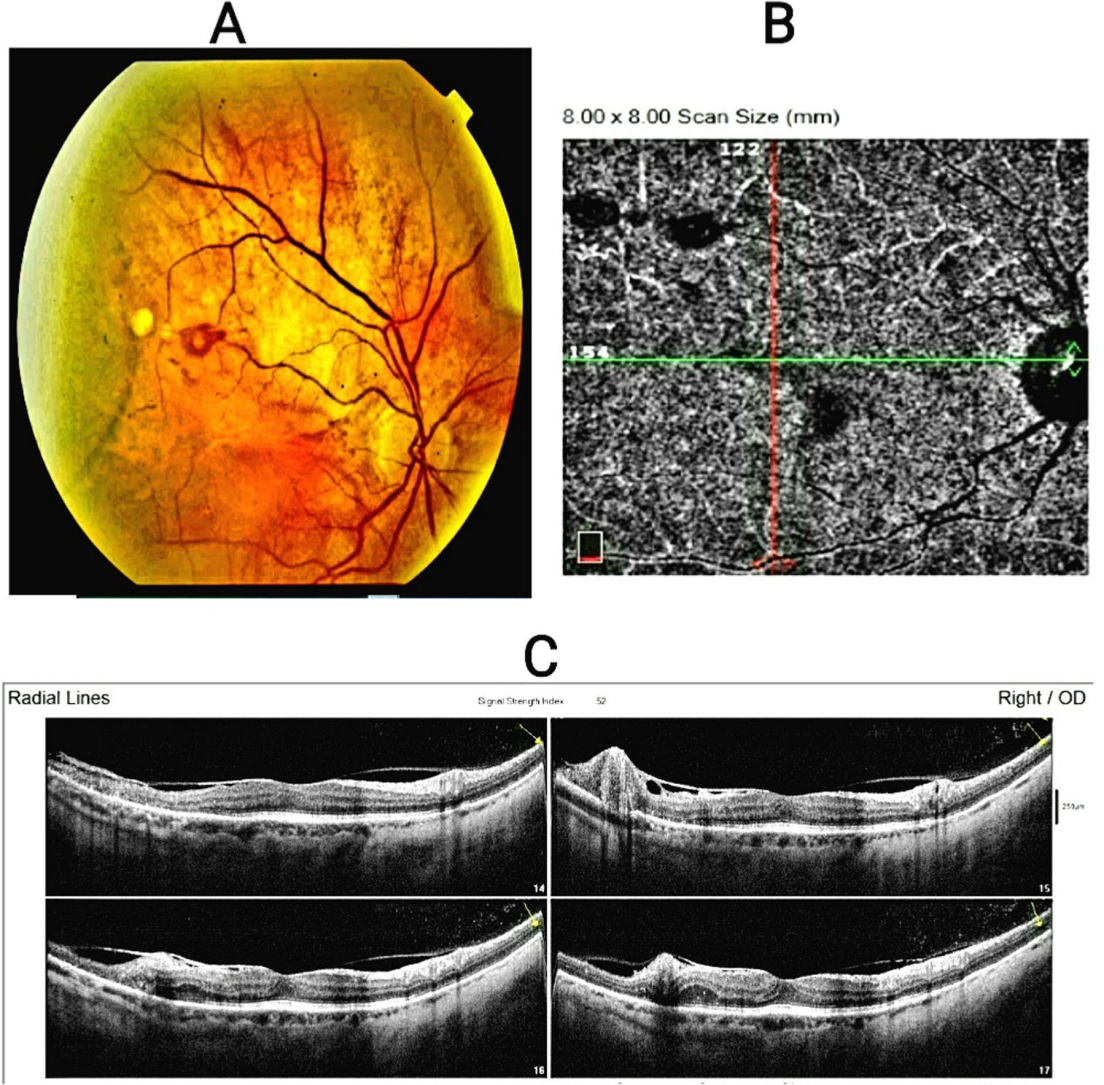
Composite images of fundus photography (**A**), OCTA (**B**), OCT (**C**) showing the 3 RAMs lesions. Fundus photograph showed hemorrhage around 3rd lesion and sclerosed other lesion along the superotemporal retinal arteriole (**A**). OCTA enface image 8 × 8 scan at level of choriocapillaries showed the RAMs lesion as flow void (**B**). OCT images radical lines scan showed line no. 15 passing through 1st lesion, line no 16 passing through 2nd lesion and line no 17 passing through 3rd lesion with exudation in retinal layers (**C**). Abbreviations: RAM, retinal artery macroaneurysm, OCTA: optical coherence tomography angiography, OCT: optical coherence tomography

**Fig. 5:**
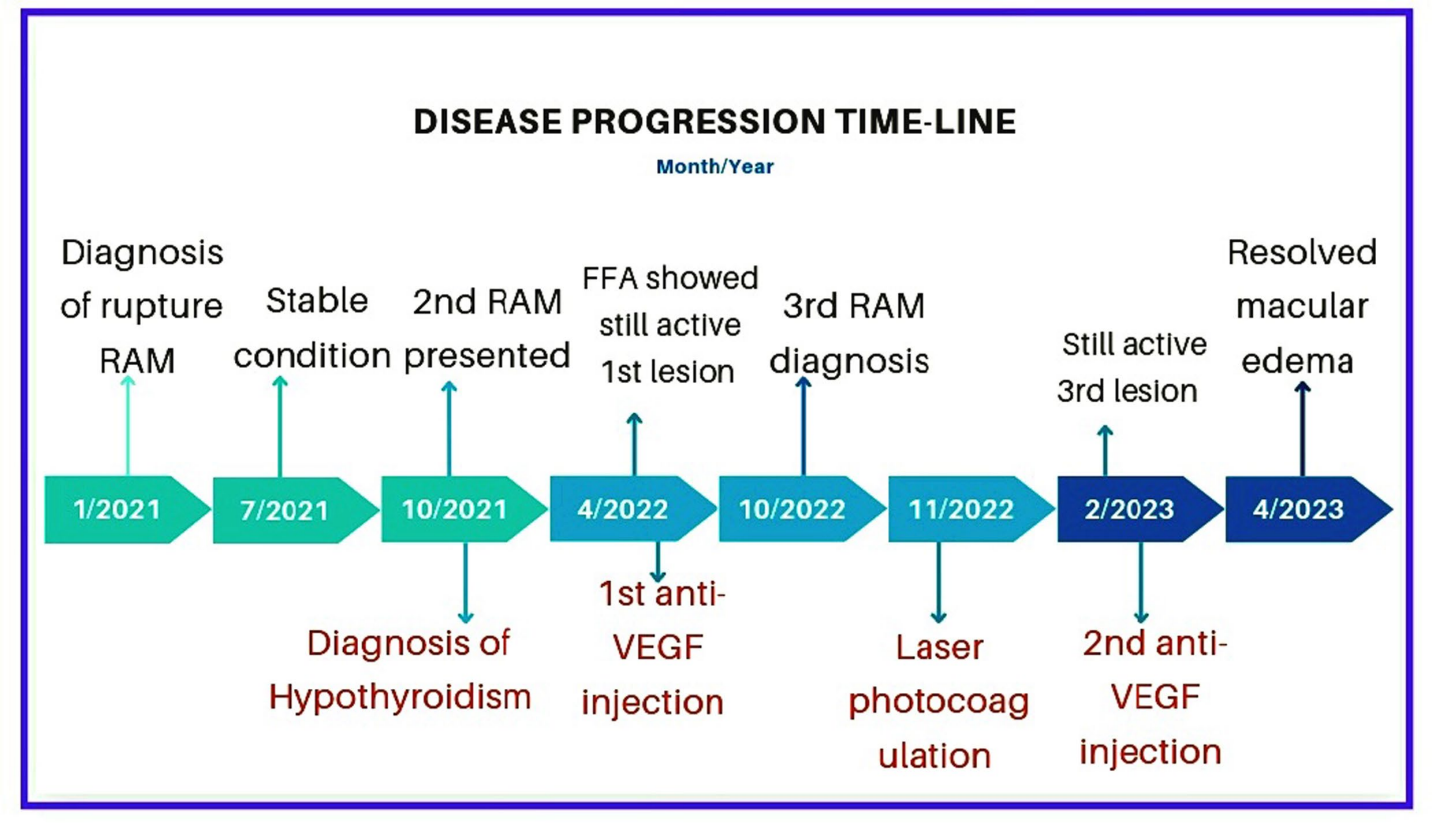
A schematic diagram showing disease progression time-line (month/year). Abbreviations: RAM, retinal artery macroaneurysm, FFA: Fundus fluorescein angiography, VEGF: vascular endothelial growth factor

Three months after getting laser photocoagulation, macular edema was still present, and cystic changes had started to develop. Repeated laboratory testing revealed a managed hypothyroid state with TSH = 1.26 uIU/ml and free T4 = 14.66 poml/l, but microalbuminuria was also present with an albumin/creatinine ratio (ACR) = 265 mg/g and within normal glycated hemoglobin A1c (HBA1C = 4.3%). Anti-VEGF (ranibizumab; LUCENTIS®) was administered a second time. After two months, the edema went away and the third lesion on the OCTA had regressed **(Supplementary Fig. 4**). The first lesion was hemorrhagic, with vitreous and retinal hemorrhages, while the second and third were mostly exudative, with minimal vitreous hemorrhage. OCT could detect various RAM sizes and levels **(**Fig. 4**).** A timeline of disease progression is displayed in **(**Fig. 5**).**

## Discussion

The most important risk factors for RAM are chronic systemic hypertension (75% of patients), arteriosclerosis, abnormal lipid levels, and inherent structural defects in blood vessels. These cause focal ischemia of the blood vessel wall, resulting in increased vessel permeability and intimal collagen remodeling[5].

RAM is characterized by a weakness in the muscularis layer of the arteriolar wall, which results in artery thinning, fibrosis, and decreased elasticity. This predisposes the artery to aneurysmal arterial dilatation. The aneurysmal dilatation is fragile and can rupture, resulting in hemorrhage and exudation of proteinaceous and lipid-rich exudates in any layer of the retina, resulting in visual loss[3].

There are different lines for managing RAMs. A spontaneous involution usually occurs in 8–25% of RAMs. A visual acuity better than 20/40 can still be maintained in 37% of patients with submacular hemorrhage without treatment [4]. However, one-third of untreated RAMs cases may experience vascular leakage and retinal edema, which call for medical or surgical intervention [4].

For the treatment of vitreous hemorrhage, oral anti-edematous enzyme is frequently employed. In current case, anti-edematous enzyme performed chemical hyaloidotomy and dissolved the subretinal hemorrhage before being stopped to avoid rebleeding and interfering with RAM spontaneous thrombosis.

In our situation, repeated anti-VEGF injections were effective in curing macular edema. Inhibiting VEGF may prevent angiogenesis, reduce binding of the prothrombotic VEGF receptor 2, and improve vascular permeability. Nonetheless, laser photocoagulation is still a viable treatment option for macular edema brought on by RAM, and the potential advantages of anti-VEGF therapy must be carefully evaluated against the risks of infection from repeated injections [4].

The most common hemodynamic effect of hypothyroidism is bradycardia, which is accompanied by mild hypertension and a narrowed pulse pressure [6].An experimental study found significant changes in the heart and blood vessels in medically induced hypothyroidism, such as endothelium enlargement and thickening of the vessel lining, hyperplasia, inflammatory cell infiltration, and fatty vacuoles in tissue cells [7].Acquired ichthyosis, often known as eczema craquelé, is one of the unusual cutaneous manifestations of hypothyroidism. Lack of thyroid hormones leads to abnormal epidermal lipid metabolism, which may contribute to acquired ichthyosis-associated hypothyroidism [8].

After reviewing previous cases of multiple RAMs, we discovered that they could appear concurrently or sequentially [9, 10]. RAMs evolve through a dynamic process of formation, enlargement, and involution. Different RAMs have distinct rupture mechanisms and display patterns. In a long-term follow-up instance, Terubayashi et al. observed unilateral numerous RAMs with divergent courses, and one of the ruptured RAMs resulted in branch retinal vein occlusion[11].RAMs lesions followed one another in current case, and each one had different criteria for size, level, and regression. RAMs’ criteria appear to reflect blood pressure control and the state of the vascular tree. Recognizing and addressing systemic linkages will reduce the possibility of enduring more RAM ruptures [11].

In educational platforms, a single RAM lesion was observed in elderly females with hypothyroidism with [12] and without associated systemic illness [13]. Multiple RAMs, on the other hand, had been recorded in young patients homozygous for the retinal arterial macroaneurysms with supravalvular pulmonic stenosis (RAMSVPS) syndrome causative IGFBP7 variant, two of whom had an associated hypothyroidism [14].

Chronically high blood pressure causes the sclerotic phase of hypertensive retinopathy, which is clinically characterized by vascular attenuation, enhanced arteriolar light reflex, arteriovenous nicking, and increased tortuosity of arterioles. Long-term consequences such macroaneurysms could result from sclerosing alterations [15]. Hypothyroidism is typically accompanied by a mild increase in blood pressure. In current case, a blood pressure chart revealed that the patient’s arterial blood pressure fluctuated, but was mostly in the mild hypertension range. The occurrence of RAM lesion in our patient preceded the diagnosis of hypothyroidism and its frequency matched dermatological hypothyroidism manifestations. Moreover, the RAM weaning was more closely related to hypothyroidism control. This raises the question of whether multiple RAM lesions and concomitant cardiovascular comorbidities in the current case were caused by hypertension, hypothyroidism, or both. The presence of microalbuminuria, or a high albumin-creatinine ratio (ACR) in people with subclinical hypothyroidism indicates a higher risk of cardiovascular mortality compared to a subclinically hypothyroid person with a low ACR [16]. In contrast, in female patients with hypertension who did not have any coexisting comorbidities, microvascular disease was linked to both a lower and a higher ACR [17]. Although our patient’s renal function was normal, she had a high ACR (265 mg/ml), which was also associated with the emergence of several RAMs lesions and macular edema. Moreover, a fundus examination of the left eye, the healthy eye, did not reveal any noteworthy alterations linked to hypertensive retinopathy other than mild sclerotic changes. Such evidences may imply that there are multiple factors influencing the vascular tree’s integrity. RAM lesion may form predominantly as a result of sclerotic alterations, but the behavior, multiplicity, and concomitant macular edema may be connected to other variables as well, such as hypothyroidism-induced altered metabolism and developing nephropathy.

## Conclusion

RAMs are typically solitary lesions, but the presence of multiple or recurring lesions should raise concerns about underlying systemic illness. Severe hypothyroidism is a potentially fatal condition, and this case report may indicate a link between it and multiple or recurring RAMs. OCT and OCTA are useful tools for RAM confirmation and follow-up. RAM treatment could be tailored to their type and location in relation to the macula. A variety of underlying hemodynamic variables may contribute to the disruption of the blood-retinal barrier and vascular integrity that causes persistent macular edema and retinal hemorrhage.

## Electronic supplementary material

Below is the link to the electronic supplementary material.

Supplementary Material 1

Supplementary Material 2

Supplementary Material 3

Supplementary Material 4

Supplementary Material 5

## Data Availability

This published article includes all data generated or analyzed during the current study.
