# Supplementary figures and images for: Triple retinal arterial macroaneurysms in a hypertensive patient with hypothyroidism
Source: BMC Ophthalmol. 2023 May 10;23:207. doi: 10.1186/s12886-023-02953-x (PMC10170779; doi:10.1186/s12886-023-02953-x)

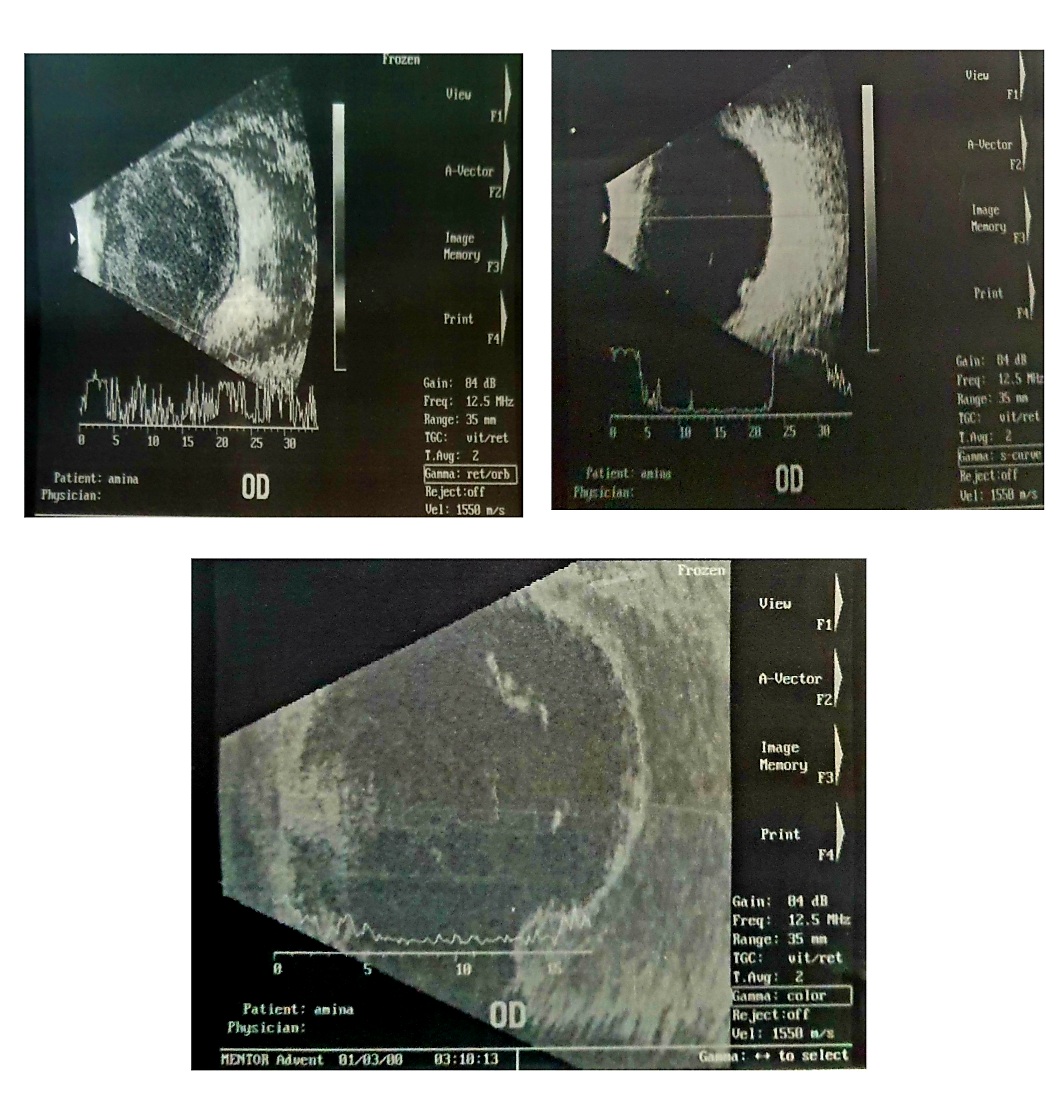

Supplement: Supplementary file 1 — Supplementary Material 1 [file 12886_2023_2953_MOESM1_ESM.jpg]

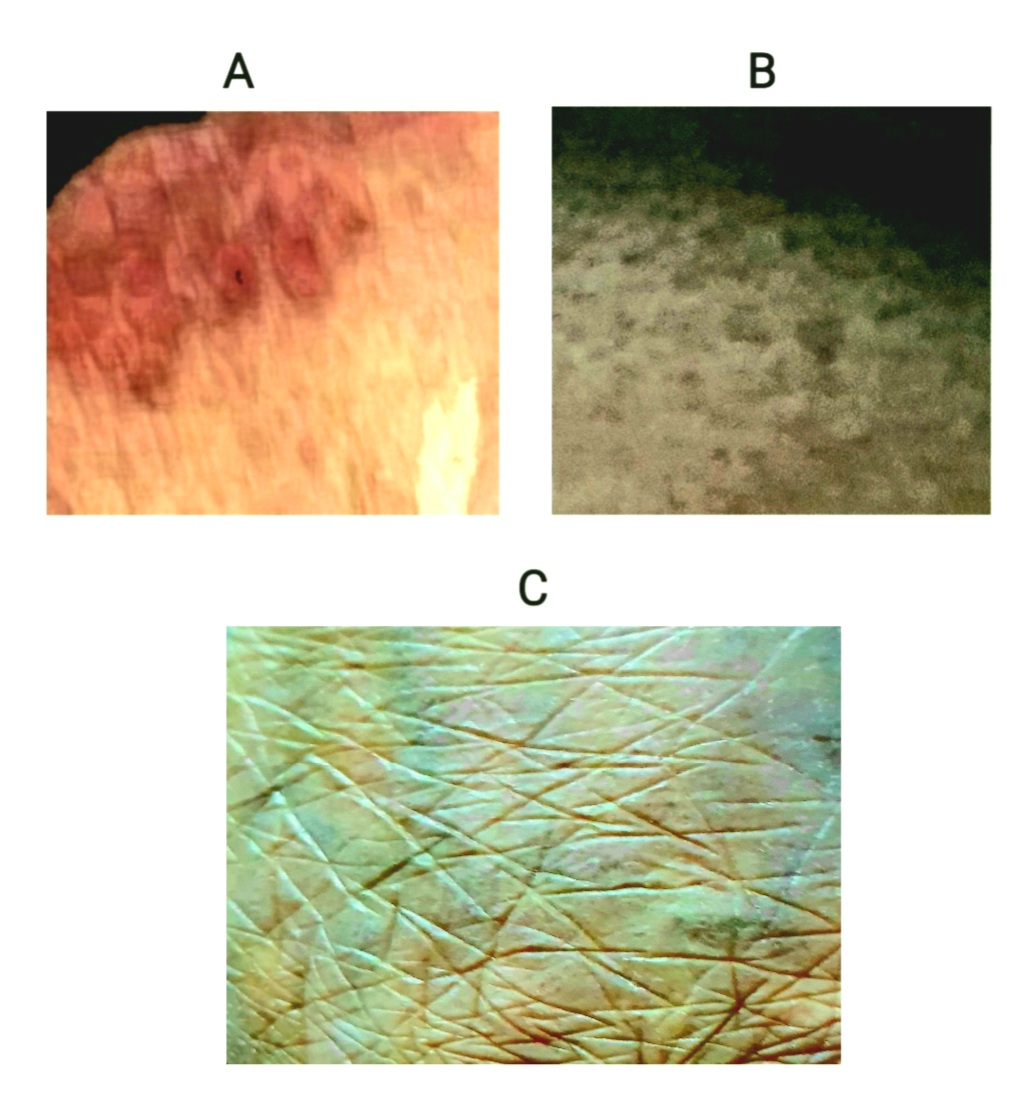

Supplement: Supplementary file 2 — Supplementary Material 2 [file 12886_2023_2953_MOESM2_ESM.jpg]

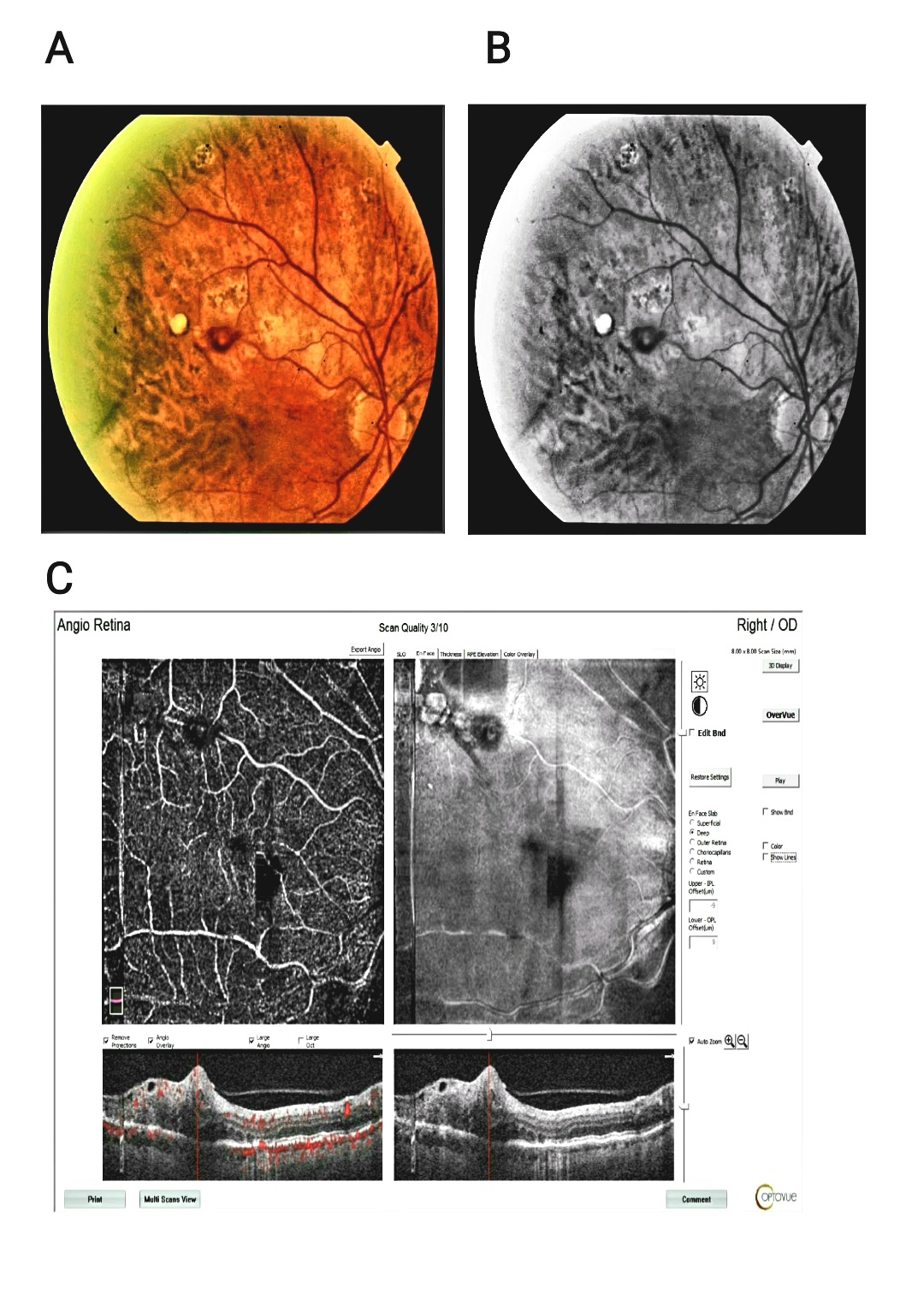

Supplement: Supplementary file 3 — Supplementary Material 3 [file 12886_2023_2953_MOESM3_ESM.jpg]

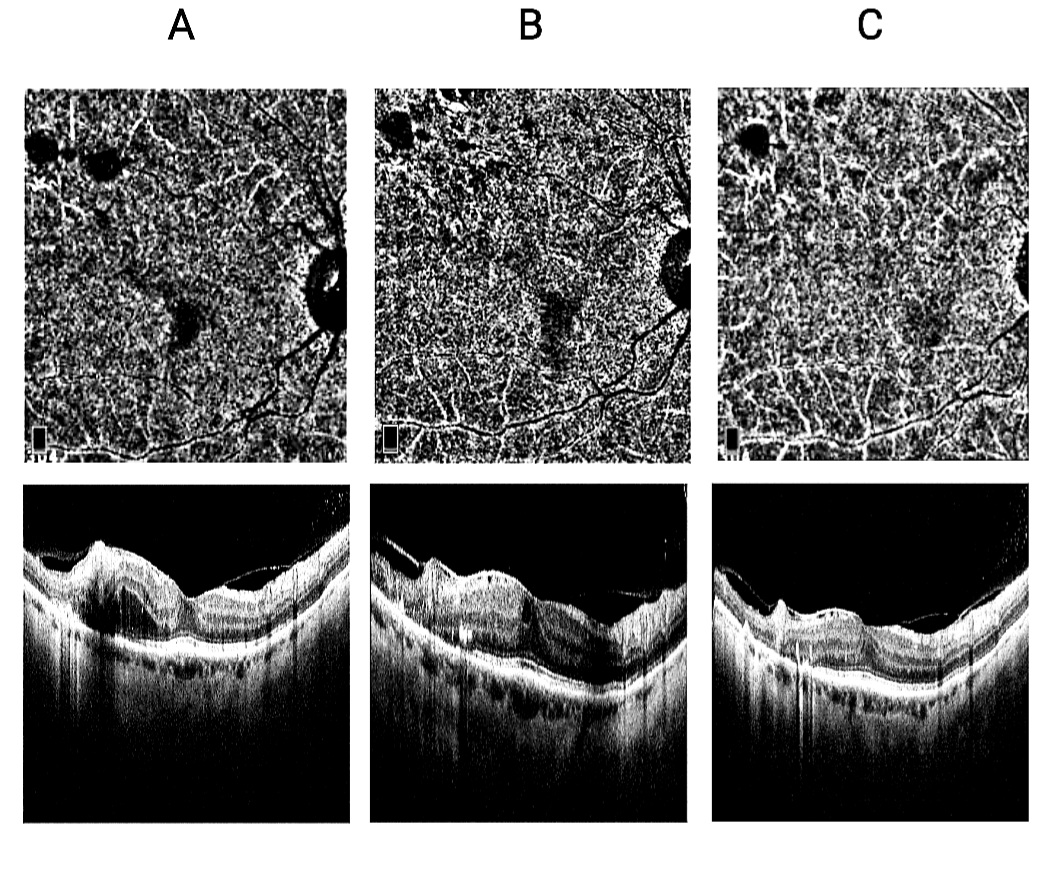

Supplement: Supplementary file 4 — Supplementary Material 4 [file 12886_2023_2953_MOESM4_ESM.jpg]
