# Supplementary material for: Triple retinal arterial macroaneurysms in a hypertensive patient with hypothyroidism
Source: BMC Ophthalmol. 2023 May 10;23:207. doi: 10.1186/s12886-023-02953-x (PMC10170779; doi:10.1186/s12886-023-02953-x)
Supplement: Supplementary file 5 — Supplementary Material 5 [file 12886_2023_2953_MOESM5_ESM.docx]

**Supplementary Figures legends**

**Supplementary Figure 1:** Ultrasonography b scan photos demonstrating vitreous hemorrhage and subretinal hyperechoic lesion corresponding to subretinal hemorrhage.

**Supplementary Figure 2:** photos showing skin lesions of acquired ichthyosis on forehead before (a) and after treatment of hypothyroidism (b) and dorsum of hand (c).

**Supplementary Figure 3:** Composite images of fundus photography (A), red free (B) OCTA(C) one week after argon laser photocoagulation.

**Supplementary Figure 4:** Composite images of OCTA (upper row), and OCT B scan (bottom row) showing the RAMs lesions.  The RAMs lesions were visible as a void in the flow on an OCTA enface image 8×8 scan at level of choriocapillaries showed the RAMs lesion as void in the flow (projection artifacts from overlaying superficial retinal layer).  After emergence of the third lesion, OCTA revealed three RAMs with associated macular edema in the OCT B scan (A). After 3 months of laser photocoagulation, imaging revealed persistent edema and the development of cystic alterations B). After 2 months of the second anti-VEGF injection, imaging shows regression of the third RAM on OCTA and improved macular edema (C).
